# Bedside Tested Ocular Motor Disorders in Multiple Sclerosis Patients

**DOI:** 10.1155/2014/732329

**Published:** 2014-04-30

**Authors:** G. Servillo, D. Renard, G. Taieb, P. Labauge, S. Bastide, M. Zorzon, G. Castelnovo

**Affiliations:** ^1^Department of Medical and Health Sciences, University of Trieste, 34100 Trieste, Italy; ^2^Service de Neurologie, Centre Hospitalier Universitaire Caremeau, 30029 Nîmes, France; ^3^Service de Neurologie, Centre Hospitalier Universitaire Guy de Chauliac, 34090 Montpellier, France; ^4^Service de Epidemiologie, Centre Hospitalier Universitaire Caremeau, 30029 Nîmes, France; ^5^UCO di Neurologia, Ospedale di Cattinara, Strada di Fiume 447, 34149 Trieste, Italy

## Abstract

*Background/Aims*. Ocular motor disorders (OMDs) are a common feature of multiple sclerosis (MS). In clinical practice, if not reported by patients, OMDs are often underdiagnosed and their prevalence is underestimated. *Methods*. We studied 163 patients (125 women, 76.7%, 38 men, 23.3%; median age 45.0 years; median disease duration 10 years; median EDSS 3.5) with definite MS (*n* = 150, 92%) or clinically isolated syndrome (*n* = 13, 8%) who underwent a thorough clinical examination of eye movements. Data on localization of previous relapses, MS subtype, and MRI findings were collected and analyzed. *Results*. Overall, 111/163 (68.1%) patients showed at least one abnormality of eye movement. Most frequent OMDs were impaired smooth pursuit (42.3%), saccadic dysmetria (41.7%), unilateral internuclear ophthalmoplegia (14.7%), slowing of saccades (14.7%), skew deviation (13.5%), and gaze evoked nystagmus (13.5%). Patients with OMDs had more severe disability (*P* = 0.0005) and showed more frequently infratentorial MRI lesions (*P* = 0.004). Localization of previous relapses was not associated with presence of OMDs. *Conclusion*. OMDs are frequent in patients with stable (no relapses) MS. A precise bedside examination of eye motility can disclose abnormalities that imply the presence of subclinical MS lesions and may have a substantial impact on definition of the diagnosis and on management of MS patients.

## 1. Introduction

Multiple sclerosis (MS) patients often experience ocular motor disorders (OMDs) during the course or sometimes as an early manifestation of the disease [1]. The most common OMDs are internuclear ophthalmoplegia (INO), disturbances of conjugate gaze, such as saccadic dysmetria and impaired smooth pursuit, gaze-evoked nystagmus, and vestibuloocular reflex (VOR) abnormalities [2–4]. Other unusual, more complex, ocular motor disturbances have also been described [5].

Eye movement accuracy is under control of many integrated components of the central nervous system, especially those located in critical areas of the brainstem and the cerebellum [6]. The widespread extension of MS lesions and the frequent involvement of infratentorial structures explain why OMDs are so common in MS patients.

The importance of an accurate examination of ocular movements is underlined by the potential impact of OMDs on disease progression and the value of OMDs as predictors of disability in MS [7, 8].

From the patient's point of view, major symptoms of OMDs are blurring of vision, oscillopsia, and diplopia, although less obvious symptoms, such as feeling of unsteadiness, may be the only complaints. Many patients, however, do not report any symptom at all [2, 9].

An accurate clinical bedside ocular motor examination, paying attention particularly to the dynamic characteristics of ocular saccades and VOR, performed by an experienced investigator, can detect the majority of OMDs [3, 4, 10]. Oculographic techniques, such as video oculography [11, 12], and electrooculography (EOG) [13, 14] can increase the precision of clinical detection.

Although MRI often can precisely delineate the lesion responsible of OMDs [15], the relationship between disability and lesion burden and the clinicoanatomical correlation of clinical signs/symptoms and the location of MS lesions are not well established [16]. The sensitivity of MRI in revealing small lesions in the brainstem is limited when the lesion size is below the threshold of detection [17], further emphasizing the importance of careful clinical examination [18].

In clinical practice, if not reported by patient, OMDs are often underestimated [9]. Data on the exact prevalence of OMDs in MS patients are rare, resulting from few small sample size studies reporting prevalence varying from 80% [19] to 32% [13] in clinically definite MS.

In patients in stable phase of disease (no relapses) who do not report symptoms of abnormal eye motor function, the detection of a silent lesion by an accurate bedside neuroophtalmologic examination may influence clinical management and therapeutic decisions, and, in the early phase of disease, contribute to the definition of the diagnosis of MS by means of the detection of a dissemination in space of the lesions [20, 21].

The aim of our study was to assess the prevalence of OMDs in a relatively large sample of patients with stable MS (i.e., in absence of acute relapse) and to look for possible correlations between the presence of OMDs and the disability at the moment of examination, a positive history of brainstem/vestibulocerebellar relapses, and the evidence of MRI lesions in the posterior fossa.

## 2. Patients and Methods

From 1 January to 30 June 2012, 195 consecutive patients with definite MS or clinically isolated syndrome (CIS) [21] were referred to the Neurological Department of Centre Hospitalier Universitaire Caremeau, Nîmes, France. Thirty-two patients were excluded because of an acute ongoing relapse or because of recent onset oscillopsia or diplopia. The remaining 163 patients were enrolled in the study (Table 1) and underwent an accurate clinical examination of eye movements by two experienced, independent investigators (G. Castelnovo and G. Servillo). In case of disagreement, a third investigator (D. Renard) performed an independent examination.

Inclusion criteria were definite diagnosis of MS or CIS according to the diagnostic criteria established by Polman et al. [21] and the age of 16–80 years. Exclusion criteria were acute relapse, concomitant treatment with corticosteroids, and recent onset of ocular motor symptoms.

The visual disability of the patients at study was low or negligible; therefore all patients had a visual acuity sufficient to perform a reliable bedside test examination of ocular movements.

We looked out for disturbances of gaze shifting, gaze holding, and ocular alignment. The clinical diagnosis of INO was defined when, in bedside examination tests, paralysis or paresis of adduction of the eye or slowed adduction saccade was present during the horizontal duction.

All included patients gave written informed consent and the study was approved by the local ethics committee.

The bedside clinical examination of eye movements was comprised of (1) examination of the eyes in primary position, (2) examination of horizontal and vertical conjugate gaze, (3) examination of pursuit ocular movements, (4) study of horizontal and vertical saccades, (5) horizontal and vertical VOR test, and (6) testing of eye convergence.

To avoid recall bias, changes in cognitive capabilities or failure to recall ocular symptoms of low magnitude, information on the clinical localization of previous relapses, MS subtype, and disease duration were collected and analyzed using the data gathered in the database of the Neurological Department of Centre Hospitalier Universitaire Caremeau, Nîmes, France. Disability (assessed by EDSS [22]) was evaluated at the same time of the clinical examination of eye movements.

Data of 123 (75.5%) patients who underwent 1.5 T conventional MRI, including at least proton density imaging (PDI), T2-weighted imaging and T1-weighted imaging, and fluid-attenuated inversion recovery (FLAIR) imaging, in the three months prior to examination, were included in the MRI data analysis.

Statistical analysis was performed using Student's *t*-test and chi-square test, as appropriate. The level of significance was set at *P* < 0.05.

## 3. Results

Seventy-seven (47.2%) patients suffered from past vestibulocerebellar relapse and 61 (37.4%) had previous brainstem relapse presenting as diplopia.

Overall, 111/163 (68.1%, C.I. 95% 60.3–75.2) patients showed at least one abnormality of eye movement. Seventy-seven (47.2%) patients had more than one OMDs. OMDs were found in 61/102 (59.8%) patients with RR MS, in 33/35 (94.3%) patients with SP MS, in 11/13 (84.6%) patients with PP MS, and in 6/13 (46.1%) patients with CIS.

Table 2 shows the number, frequency, and type of OMDs found in 150 MS and 13 CIS patients.

Most frequent OMDs were impaired smooth pursuit *n* = 69 (42.3%), saccadic dysmetria *n* = 68 (41.7%), unilateral INO *n* = 24 (14.7%), slowing of saccades *n* = 24 (14.7%), skew deviation *n* = 22 (13.5%), gaze-evoked nystagmus *n* = 22 (13.5%), and VOR abnormalities *n* = 16 (9.8%).

We found a positive association between the presence of OMDs and the severity of disability. Patients with EDSS score >4.0 showed significantly more frequent OMDs than patients with EDSS ≤4 (*n* = 33/36, 91.7% versus *n* = 76/125, 60.8%; chi square *P* = 0.0005). With an EDSS cut-off score of 2.0, we found the same result (*n* = 96/130, 73.8% versus 13/31, 41.9%; chi square *P* < 0.0001).

Eighty-four (68.3%) of the analyzed patients on MRI had at least one lesion in the brainstem and/or cerebellar regions. Infratentorial MRI lesions were found in 63/82 (76.8%) patients with OMDs and in 21/41 (51.2%) patients without OMDs (chi square *P* = 0.004).

We failed to find an association between OMDs and past history of clinical vestibulocerebellar (chi square *P* = NS) or brainstem (chi square *P* = NS) relapses.

## 4. Discussion

In the present study, we demonstrated that bedside ocular movements examination, in a relatively large (*n* = 163) sample of patients with MS and CIS, reveals a high number of OMDs. More than 2/3 of patients showed at least one type of OMDs, and almost half of the patients had more than one type of OMDs.

A comparison with previous studies reporting on prevalence of OMDs in MS patients is difficult because of differences in sample size, characteristics of patients, diagnostic criteria, and methods of ascertainment. However, our findings seem to confirm earlier reported data by Reulen et al. who described the presence of subclinical eye movement disorders in 80% of definite MS patients [19]; Knezevic et al. who reported the presence of one or more, often subclinical, abnormal saccade parameters in 67% of patients with clinically definite, probable or suspected MS [23]; Muri and Meienberg who observed one or more OMDs in 76% of systematically examined patients with definite MS, using simple clinical tests verified on healthy controls [24]. Lower prevalence of OMDs was reported by other studies. For instance, Tsuda et al. observed ocular motor symptoms in only 36% of patients with MS examined by neuroophtalmologists [25], and Jozefowicz-Korczynska et al. found clinical eye movement disorders in 32% of patients with MS [13]. Although the latter authors, in a study aimed to determine the frequency of smooth pursuit disturbances in MS patients, found disorders in clinical bedside smooth pursuit test examination in 25% of MS patients, they detected subclinical smooth pursuit disturbances in 76.6% of cases with EOG [14].

In our cohort of MS patients we found a positive association between OMDs and disability assessed by EDSS. In more disabled patients, the prevalence of OMDs was significantly higher (*P* ranging from <0.0001 to = 0.0005 according to increasing severity of EDSS score). These findings confirm data reported by Serra et al. who, using bedside examination, showed that MS patients with abnormalities of eye movements had more severe disability than those without OMDs [7]. At 2-year follow-up patients with abnormal eye movements have been described to show greater progression of EDSS score, demonstrating that the presence of OMDs may predict disability in MS [8]. Also another recent study demonstrated that pursuit system impairment correlates with the degree of neurological disability [14].

We also found an association between the presence of MS lesions in the posterior fossa (as evidenced by conventional MRI) and OMDs (*P* = 0.004). More than half of our patients with MRI brainstem and/or cerebellum lesions had normal eye movement examination (false positive), whereas almost a quarter of patients with OMDs had normal MRI (false negative). Frohman et al. studied the characteristics of conventional MRI of the medial longitudinal fasciculus (MLF) in 58 patients with chronic INO and found a MLF lesion hyperintensity on PDI, T2-weighted images, and FLAIR images in 100%, 88%, and 48% of patients, respectively [15]. However, since they included neither patients with other forms of OMDs nor patients without abnormalities of eye movements, the specificity and the sensitivity of conventional MRI in patients with OMDs could not be established.

Finally, we did not find an association between records of previous vestibulocerebellar and/or brainstem relapses and OMDs (*P* = NS), further emphasizing the possibility of subclinical lesions and/or the lack of awareness by patients for some aspects of visual function.

We conclude that OMDs are frequent in MS patients even in stable phase of disease. OMDs may not be noticed or reported by patients, explaining the probable underestimation of the true prevalence of OMDs when accurate bedside examination is not performed. A precise bedside examination of eye motility can disclose abnormalities that imply the presence of subclinical MS lesions that may have a substantial impact not only on the diagnosis of MS with the definition of a dissemination in space of the lesions [21] but also on the clinical management of MS patients [14].

## Figures and Tables

**Table 1:** Characteristics of patients.

Gender *n* (%)	Females 125 (76.7)	Males 38 (23.3)
Age	Mean (SD) 44.9 (12.8) years	Median 45.0 years
EDSS	Mean (SD) 3.4 (1.8)	Median 3.5
Disease duration	Mean (SD) 11.9 (9.5) years	Median 10 years
Type of disease *n* (%)	RR 102 (62.6)	
	SP 35 (21.5)	
	PP 13 (8)	
	CIS 13 (8)	

EDSS: Expanded Disability Status Scale [22]; RR: relapsing-remitting; SP: secondary-srogressive; PP: primary-progressive; CIS: clinically isolated syndrome.

**Table 2:** Ocular motor disorders in 150 patients with definite multiple sclerosis and 13 patients with clinically isolated syndrome.

Ocular motor disorders	No.	%
Impaired smooth pursuit	69	42.3
Saccadic dysmetria	68	41.7
Unilateral internuclear ophthalmoplegia	24	14.7
Slowing of saccades	24	14.7
Skew deviation	22	13.5
Gaze-evoked nystagmus	22	13.5
Pathological vestibuloocular reflex	16	9.8
Impaired convergence	10	6.1
Upbeat nystagmus	9	5.5
Bilateral internuclear ophthalmoplegia	8	4.9
Upward gaze palsy	5	3.1
Ocular nerve palsy	4	2.4
Ocular flutter	3	1.8
Downbeat nystagmus	1	0.6
Other OMDs	4	2.4

## References

[1] Frohman EM, Frohman TC, Zee DS, Mc Coll R, Galetta S (2005). The neuro-ophtalmology of multiple sclerosis. *The Lancet Neurology*.

[2] Barnes D, Mc Donald WI (1992). The ocular manifestations of multiple sclerosis. 2. Abnormalities of eye movements. *Journal of Neurology, Neurosurgery & Psychiatry*.

[3] Niestroy A, Rucker JC, Leigh RJ (2007). Neuro-ophtalmologic aspects of multiple sclerosis: using eye movements as a clinical and experimental tool. *Journal of Clinical Ophthalmology*.

[4] Tilikete C, Jasse L, Vukusic S (2011). Persistent ocular motor manifestations and related visual consequences in multiple sclerosis. *Annals of the New York Academy of Sciences*.

[5] de Seze J, Vukusic S, Viallet-Marcel M (2006). Unusual ocular motor findings in multiple sclerosis. *Journal of the Neurological Sciences*.

[6] Pierrot-Deseilligny C, Gaymard B (1990). Eye movement disorders and ocular motor organization. *Current Opinion in Neurology and Neurosurgery*.

[7] Serra A, Derwenskus J, Downey DL, Leigh RJ (2003). Role of eye movement examination and subjective visual vertical in clinical evaluation of multiple sclerosis. *Journal of Neurology*.

[8] Derwenskus J, Rucker JC, Serra A (2005). Abnormal eye movements predict disability in MS: two-year follow-up. *Annals of the New York Academy of Sciences*.

[9] Rougier M-B, Tilikete C (2008). Ocular motor disorders in multiple sclerosis. *Journal Francais d’Ophtalmologie*.

[10] Józefowicz-Korczyńska M, Gryczyński M, Starska K, Pietruszewska W, Durko M, Lukomski M (2009). Clinical ocular-motor disturbances in Multiple Sclerosis. *Otolaryngologia Polska*.

[11] Frohman TC, Frohman EM, O’Suilleabhain P (2003). Accuracy of clinical detection of INO in MS: corroboration with quantitative infrared oculography. *Neurology*.

[12] de Santi L, Lanzafame P, Spanò B (2011). Pursuit ocular movements in multiple sclerosis: a video-based eye-tracking study. *Neurological Sciences*.

[13] Jozefowicz-Korczynska M, Łukomski M, Pajor A (2008). Identification of internuclear ophthalmoplegia signs in multiple sclerosis patients: saccade test analysis. *Journal of Neurology*.

[14] Jozefowicz-Korczynska M, Pajor AM (2011). Evaluation of the smooth pursuit tests in multiple sclerosis patients. *Journal of Neurology*.

[15] Frohman EM, Zhang H, Kramer PD (2001). MRI characteristics of the MLF in MS patients with chronic internuclear ophthalmoparesis. *Neurology*.

[16] Daumer M, Neuhaus A, Morrissey S, Hintzen R, Ebers GC (2005). MRI as an outcome in multiple sclerosis clinical trials. *Neurology*.

[17] Gass A, Filippi M, Rodegher ME, Schwartz A, Comi G, Hennerici MG (1998). Characteristics of chronic MS lesions in the cerebrum, brainstem, spinal cord, and optic nerve on T1-weighted MRI. *Neurology*.

[18] Prasad S, Galetta SL (2010). Eye movement abnormalities in multiple sclerosis. *Neurologic Clinics*.

[19] Reulen JPH, Sanders EACM, Hogenhuis LAH (1983). Eye movement disorders in multiple sclerosis and optic neuritis. *Brain*.

[20] van Dongen MM, Bertelsmann FW, Polman CH (1991). Sensitivity of eye movement registration and visual evoked potentials in evaluation of therapy in patients with multiple sclerosis. *Journal of the Neurological Sciences*.

[21] Polman CH, Reingold SC, Banwell B (2011). Diagnostic criteria for multiple sclerosis: 2010 revisions to the McDonald criteria. *Annals of Neurology*.

[22] Kurtzke JF (1983). Rating neurologic impairment in multiple sclerosis: an expanded disability status scale (EDSS). *Neurology*.

[23] Knezevic W, Mastaglia FL, Black JL, Collins DW (1984). Brainstem auditory evoked responses and quantitative saccade studies in multiple sclerosis: a comparative evaluation. *Clinical and experimental neurology*.

[24] Muri RM, Meienberg O (1987). Clinical detection of eye movement disorders in multiple sclerosis. Findings in 100 patients. *Nervenarzt*.

[25] Tsuda H, Ishikawa H, Matsunaga H, Mizutani T (2004). A neuro-ophthalmological analysis in 80 cases of multiple sclerosis. *Clinical Neurology*.

